# Mapping of the Somatosensory Cortex

**DOI:** 10.7759/cureus.7332

**Published:** 2020-03-19

**Authors:** Faisal R Jahangiri, Katharine Pautler, Keri Watters, Sahar S Anjum, Gabrielle L Bennett

**Affiliations:** 1 Neurophysiology, Axis Neuromonitoring, Richardson, USA; 2 Neurophysiology, Global Innervation, Dallas, USA; 3 Applied Cognition and Neuroscience, The University of Texas at Dallas, Richardson, USA; 4 Pre-Professional Biology, Concord University, Athens, USA; 5 Neurology, The University of Texas Health Science Center at Houston, Houston, USA; 6 Cognition and Neuroscience, The University of Texas at Dallas, Richardson, USA; 7 Brain and Behavioral Sciences Applied Cognition and Neuroscience, The University of Texas at Dallas, Richardson, USA

**Keywords:** cortical sensory mapping, sensory cortex, ssep, somatosensory evoked potentials, ionm, neurophysiology, intraoperative neuromonitoring

## Abstract

Intraoperative sensory cortical mapping is a reliable and safe method for the functional localization of the central sulcus (CS). It is utilized during neurosurgical procedures performed near eloquent brain tissue. It helps in identifying the somatosensory cortex and CS, hence preventing any postoperative neurological deficits. When executed properly, this method can identify the somatosensory cortex for both the upper and lower limbs by locating the CS. This technical report outlines the benefits of cortical sensory mapping (CsM) and detailed methodology. With the help of a properly trained intraoperative neuromonitoring staff who can accurately interpret the signals being monitored, CsM can help in injury prevention during brain surgeries.

## Introduction

The surgical procedures involving the resection of lesions located near or within the central sulcus (CS) increase the risk of postoperative neurological deficits. Motor and sensory functions in the contralateral face, upper, and lower extremities are a few of the major concerns. A multimodality intraoperative neurophysiological monitoring (IONM) with cortical sensory mapping (CsM) and cortical motor mapping (CmM) is a well-published method for the identification of the CS. The accurate identification of the CS and the eloquent tissue in the exposed cortex can decrease the risk of any postoperative neurological deficits [1]. The IONM provides more accurate functional and real-time intraoperative feedback to the surgeon as compared to preoperative magnetoencephalography (MEG) and functional magnetic resonance imaging (fMRI). The CsM is vital in assuring quality outcomes of surgeries affecting the eloquent tissue of the brain. This mapping technique is utilized to identify the CS, postcentral and precentral gyri and be able to record from the sensory cortex to ensure that the sensory pathway has not been affected by surgical practices [2].

Monitoring sensory functioning while performing a procedure on the somatosensory cortex is essential to minimize any loss of sensory function, to prevent accidental clipping of a critical vessel, and to prevent lesions to important brain regions [3]. The CsM can be utilized during the surgical procedure involving but not limited to epilepsy, arteriovenous malformations (AVM), aneurysms, embolism, and brain tumors. AVM only leads to symptomology in approximately 12% with neurological AVMs typically resulting in more adverse effects. A small portion, only 4%, of AVMs result in hemorrhage, and the risk of “steal” effect or lack of oxygen to the brain also exists. Sometimes to treat this, a surgeon may implement sclerotherapy and embolization. Both these procedures may possess a risk of neural damage. Thus, cortical mapping can reduce these risks by accurately identifying the CS, sensory, and motor cortex [4]. In surgical resection of the brain tumor, the CsM may be used to locate precentral and postcentral gyri both preoperatively and intraoperatively. This methodology allows for localization of the sensorimotor cortex via somatosensory evoked potentials (SSEP) monitoring showing a success rate of 92%. However, limitations exist in tumor resection of peri-rolandic masses [5].

## Technical report

Anesthesia

Total intravenous anesthesia (TIVA) is a preferred recommended anesthesia technique for CsM. If the patient is intubated, a train of four (TOF) monitoring should be performed from the most distal muscle.

Intraoperative neurophysiological monitoring

After intubation, the patient is placed in a supine position with the head turned either right (left side tumor) or left (right side tumor) and fixed with the operating table with pins in a Mayfield frame. Surface adhesive electrodes are placed for stimulation of upper and lower limb SSEPs. For upper limb SSEP stimulation, the electrodes were placed at the wrist for ulnar nerve (UN) and median nerve (MN), and at the medial ankle for the posterior tibial nerve (PTN).

For the MN setup, the ground is placed on the palmar surface of the forearm. The stimulating cathode is placed between the tendons of the palmaris longus and flexor carpi radialis tendons, 2 cm proximal to the crease of the wrist. The anode is placed 2-3 cm distal to the cathode on the palmar surface [6]. For the UN stimulation, the cathode is placed 2-4 cm proximal to the wrist crease on the side of the flexor carpi ulnaris. The anode is placed 2-3 cm distal to the cathode, and the ground is placed on the palmar surface of the forearm. The ground for the PTN is placed on the calf. The cathode is placed at the ankle midway between the medial border of the Achilles tendon and the posterior border of the medial malleolus. The anode is placed 2-3 cm distal to the cathode [3, 6].

The subdermal needle electrodes are placed according to the international 10-20 system guidelines for recording the upper and lower limbs SSEPs at FPz, CPz, CP3, CP4, Cv5 (fifth cervical spine), EP (left and right Erb points), and PF (popliteal fossa) [7]. The baseline SSEP should be recorded after intubation and before incision. The MN stimulation intensity: 25-30 milliamperes (mA), pulse width (PW): 300 microseconds (μs), and repetition rate (RR): 2-5 per second. The UN stimulation intensity: 15-25 mA, PW: 300 μs, and RR: 2-5 per second. The PTN stimulation intensity: 45-75 mA, PW: 300 μs, and RR: 2-5 per second. The bandpass filter includes a 30 Hz low-frequency filter (LFF) and a 3000 Hz high-frequency filter (HFF) with a sweep of 100 milliseconds and 20-50 averages.

**Figure 1:**
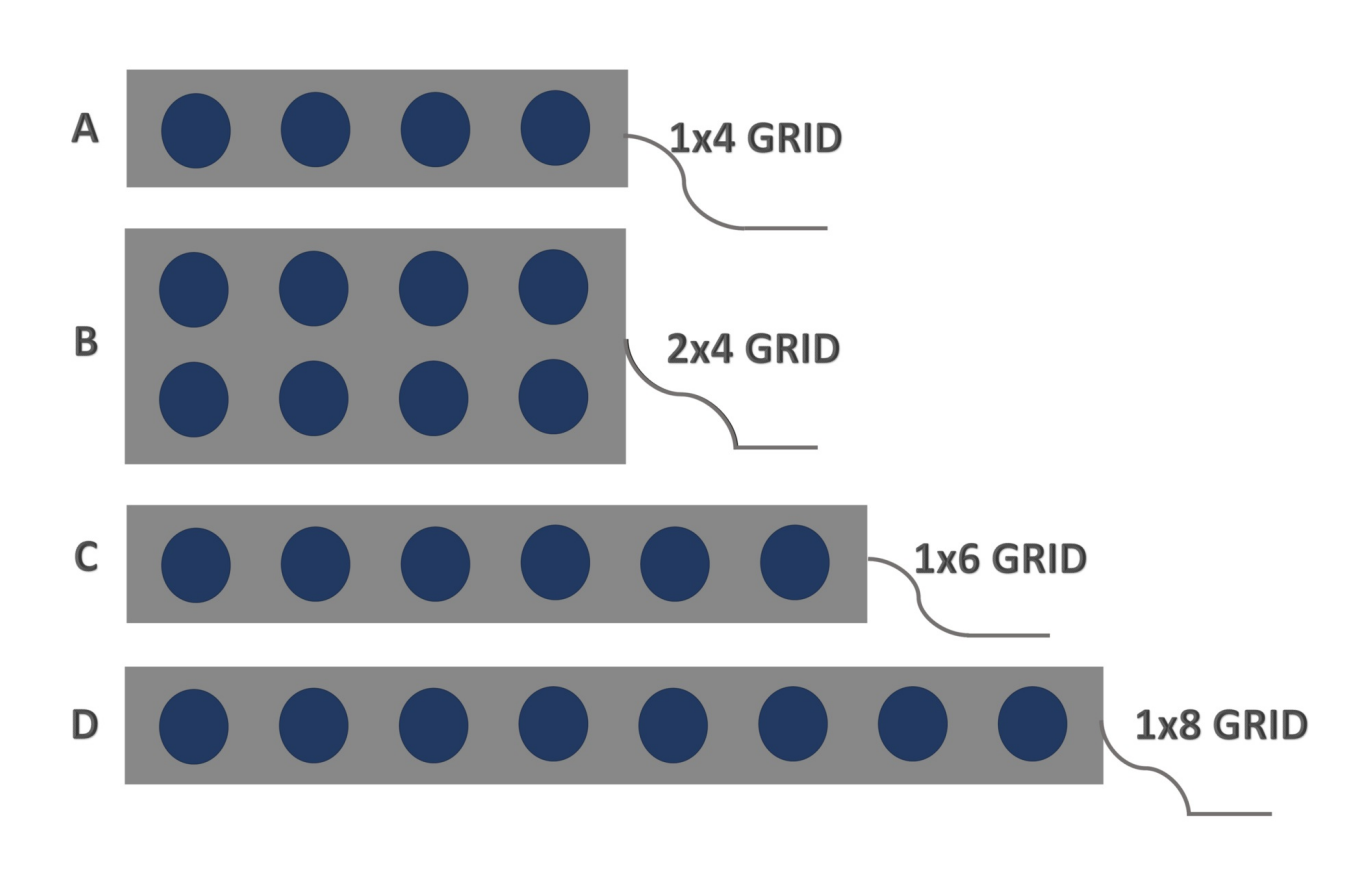
Cortical grids. Schematic presentation of the cortical grids. A: four-contact grid (1 x 4); B: eight-contact grid (2 x 4); C: six-contact grid (1 x 6); and D: eight-contact grid (1 x 8).

**Figure 2:**
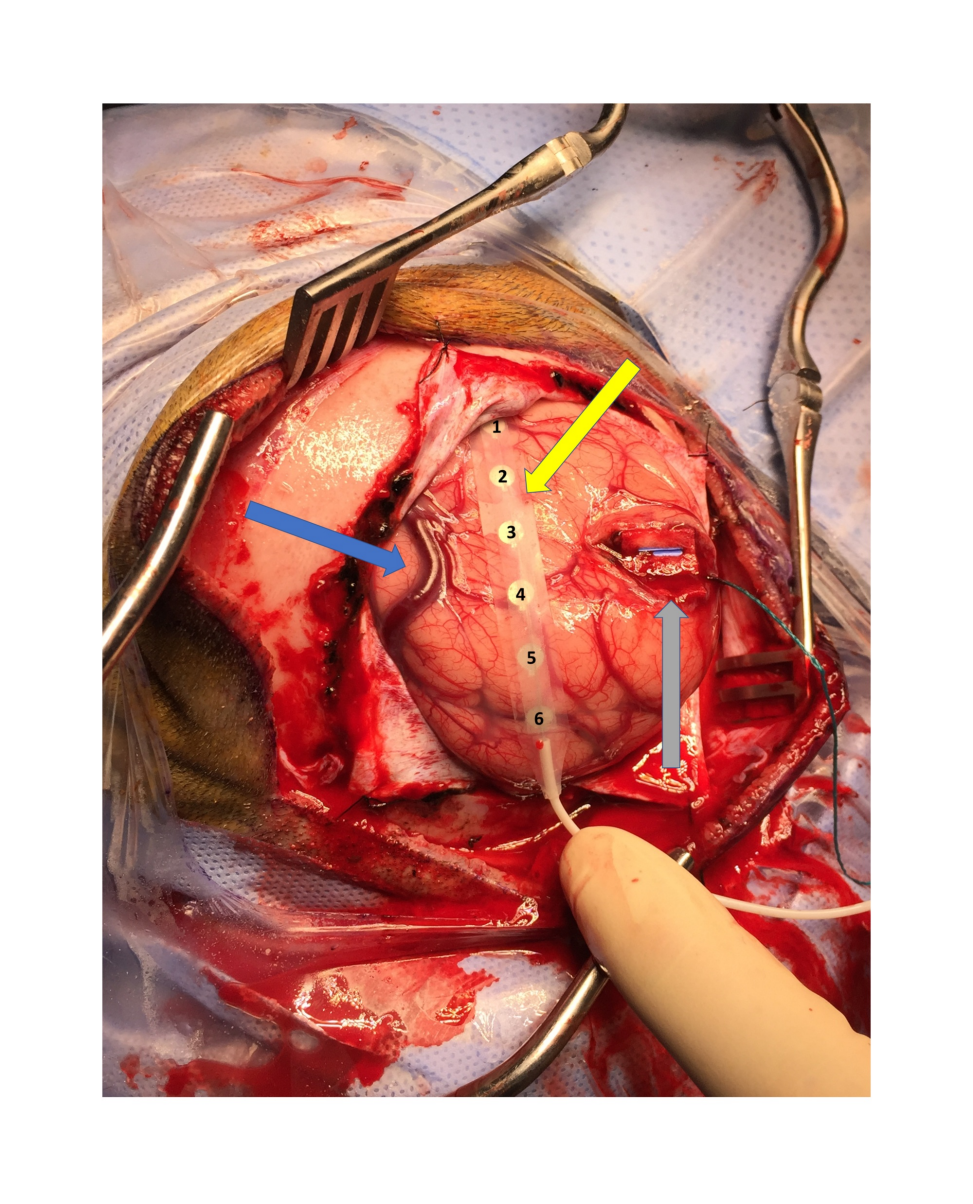
Grid placement for PR. A photograph of the exposed brain after opening the dura. A six-contact (1 x 6) grid placed on the cortex (yellow arrow), omega (blue arrow), and the tumor (gray arrow). PR, phase reversal

**Figure 3:**
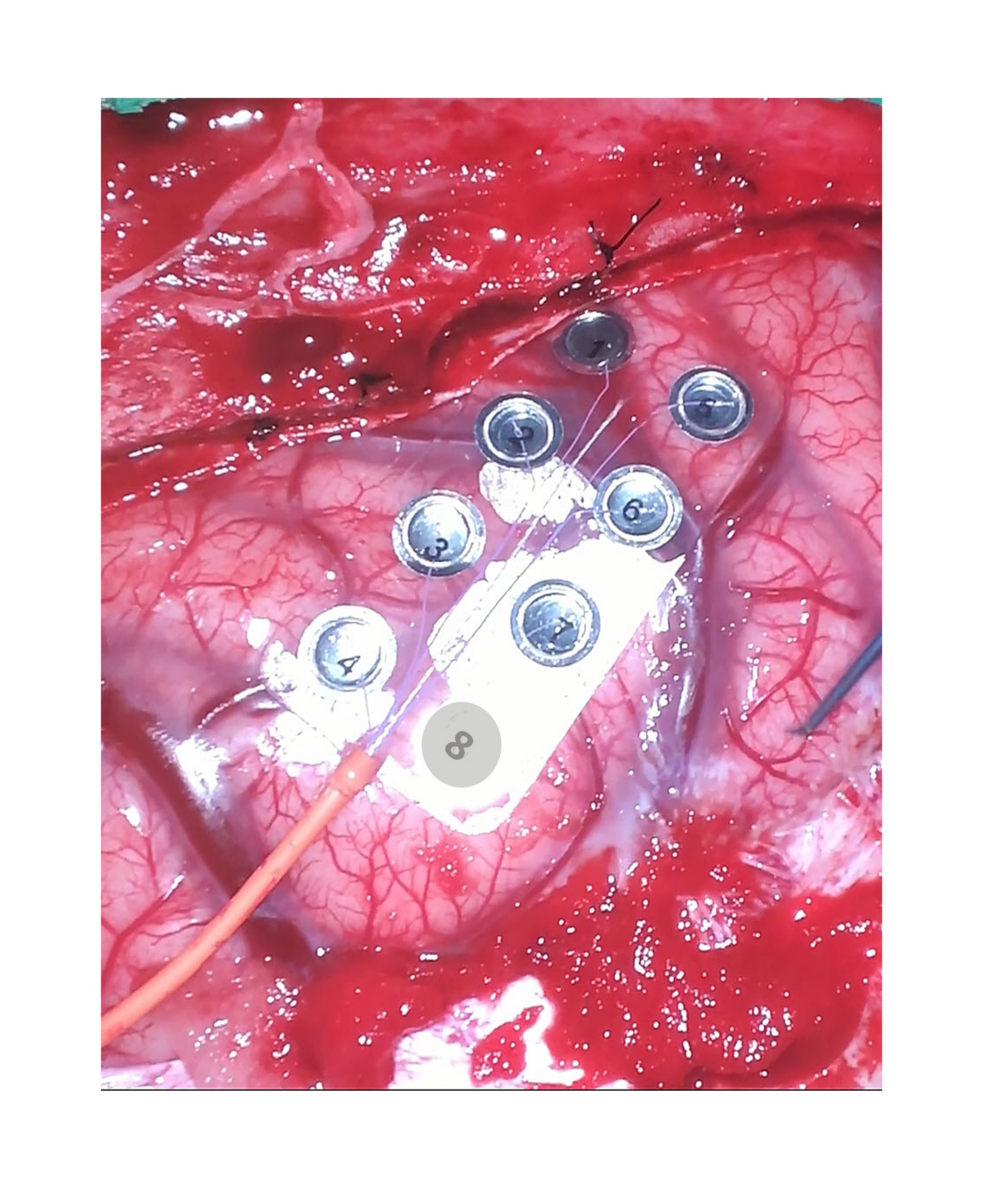
Grid placement for PR. An exposed part of the brain after opening the dura. An eight-contact (2 x 4) grid is placed on the cerebral cortex. PR, phase reversal

The CsM with phase reversal (PR) is performed by stimulating the contralateral ulnar, median, and PTN are recording from the ipsilateral cortical surface. After the cerebral cortex is exposed adequately, a subdural electrode grid made of stainless steel or platinum disc electrodes embedded in flexible silicone is placed on the cortical surface (Figures 1-3). A subdural grid with eight-contacts (2 x 4) (Figure 1) is placed where the CS is assumed to be for waveform interpretation [8]. Depending upon the location of the exposed cortex and tumor size, an alternate size grid can be placed (1 x 4, 1 x 6, or 1 x 8). The electrodes should not be floating in a pool of blood, CSF, or irrigating solution.

**Figure 4:**
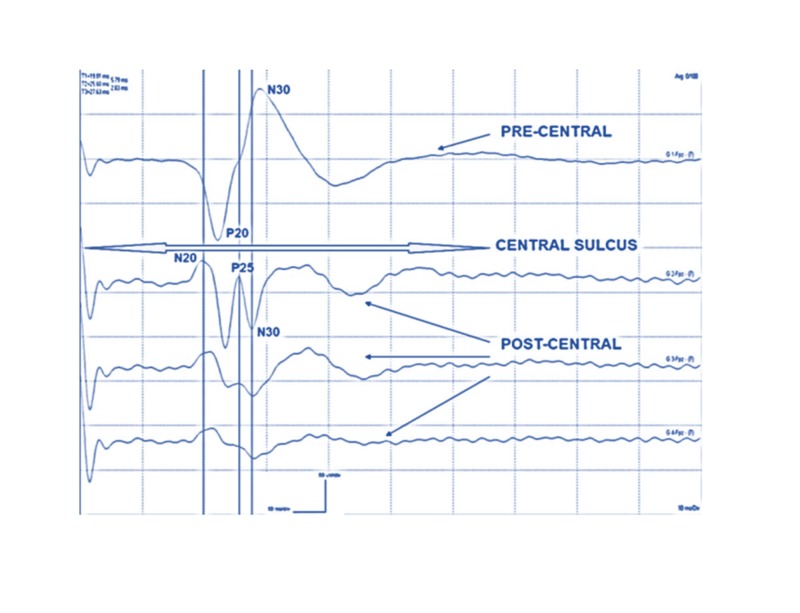
On-axis mapping with P25. On-axis MN sensory mapping by a 1x4 grid with a triphasic phase reversal, including a P25 response. The PR is between G1 and G2. The response from G1 is precentral, and G2, G3, and G4 are postcentral. MN, median nerve; PR, phase reversal

**Figure 5:**
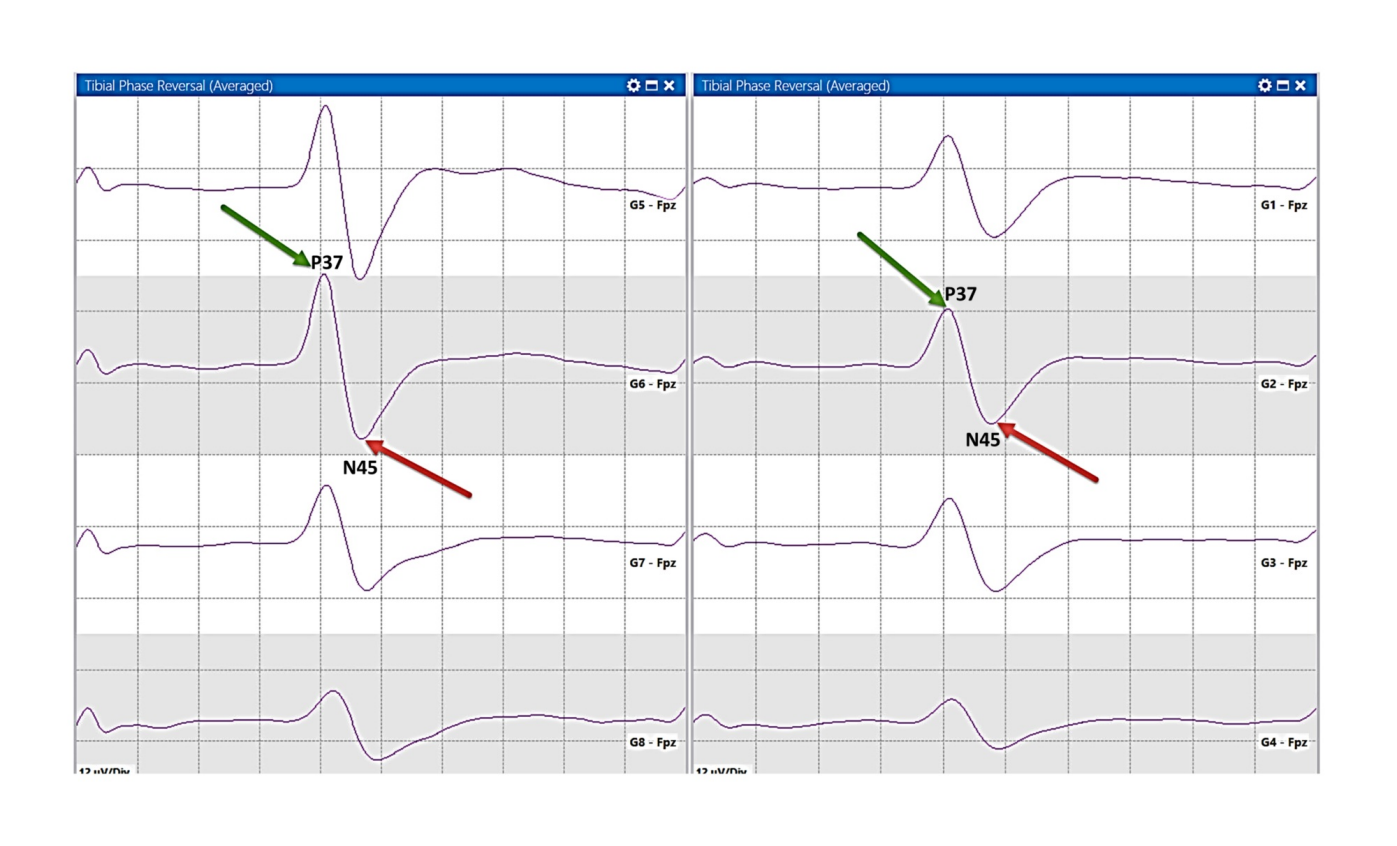
PTN sensory mapping. A PTN sensory mapping by a 2 x 4 grid. The PR is not present. The CS is localized by the largest amplitude responses  (P37 peak) from G2 and G6. A large positive P37 peak (green arrow) and a large negative N45 peak (red arrow). PTN, posterior tibial nerve; PR, phase reversal; CS, central sulcus

The referential recordings taken from grid contacts over the precentral and postcentral gyri produce a median and UN PR between the N20 and P20 (Figure 4). This PR allows the ability to locate the CS between the two contacts. If the exposed cortex is situated posterior to the CS, all the responses will be postcentral with negative upward peaks (N20). If the exposed cortex is located anterior to the CS, all the responses will be precentral with positive downward peaks (P20). If an apparent PR is not seen, then the contact with the largest amplitude of the N20 and P20 waveforms give the general location of the CS. If the tumor is near the midline close to lower limb representation on the cerebral cortex, CsM with the PTN is performed. A PR is rarely recorded for PTN. The location of the CS is either directly beneath or a few millimeters anterior or posterior to the area of the largest P37/N45 wave amplitude (Figure 5).

## Discussion

Mapping for the sensorimotor cortex is typically done for tumors located in the frontal or parietal lobes, and within or encroaching upon the sensorimotor areas. The patient is usually under general anesthesia. Dependent upon the location desired for mapping, different nerves are utilized. Upper extremities require median or UNs, whereas lower extremities rely on the PTN [9]. These procedures should be mapped using both SSEPs recorded from the postcentral sensory cortex to identify the CS, and by direct cortical motor evoked potentials (dcMEP) stimulation to identify motor cortex. The localization of the CS is the first step in identifying the precentral and postcentral gyri. If the motor gyrus is outside the tumor resection area, the further motor mapping may not be needed. The PR is a phenomenon due to the opposite polarity of the direct cortical SSEP (dcSSEP) signals recorded across the CS.

The CS is about halfway between the rostral and caudal poles of the hemispheres. This sulcus divides the frontal lobe in the rostral half of the hemisphere from the more caudal parietal lobe [10]. Utilization of CsM by stimulating contralateral MN, UN, and PTN may be used to identify the CS. The tumor could potentially push the CS, either anterior, posterior, medial, or lateral. Although the CS varies in how it physically looks from person to person, there are general landmarks that can help distinguish it from other structures surrounding it. The sigmoidal hook, where the precentral gyrus bulges posteriorly, also known as the omega sign, is a feature of the CS.

**Figure 6:**
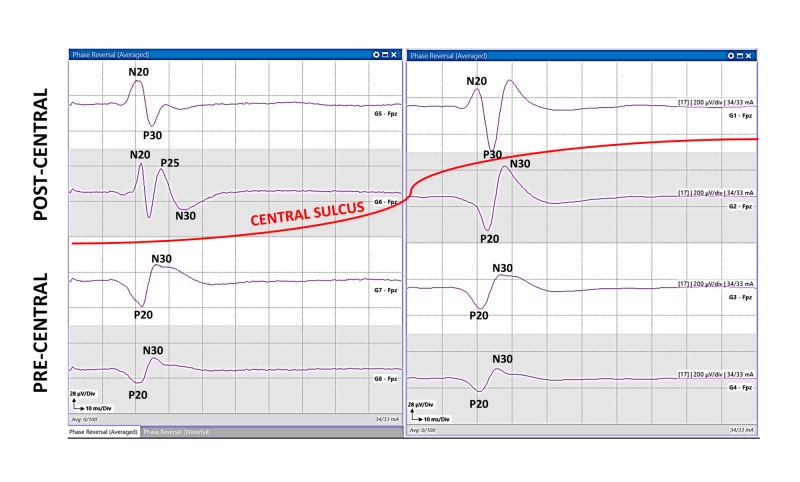
Off-axis mapping. Incomplete on-axis (off-axis) MN sensory mapping by a 2 x 4 grid with a triphasic PR, including a P25 response. The PR is between G1/G2 and G6/G7 localizing the CS (red line). The responses from G1, G5, and G6 are postcentral, and G2, G3, G4, G7, and G8 are precentral. MN, median nerve; PR, phase reversal; CS, central sulcus

**Figure 7:**
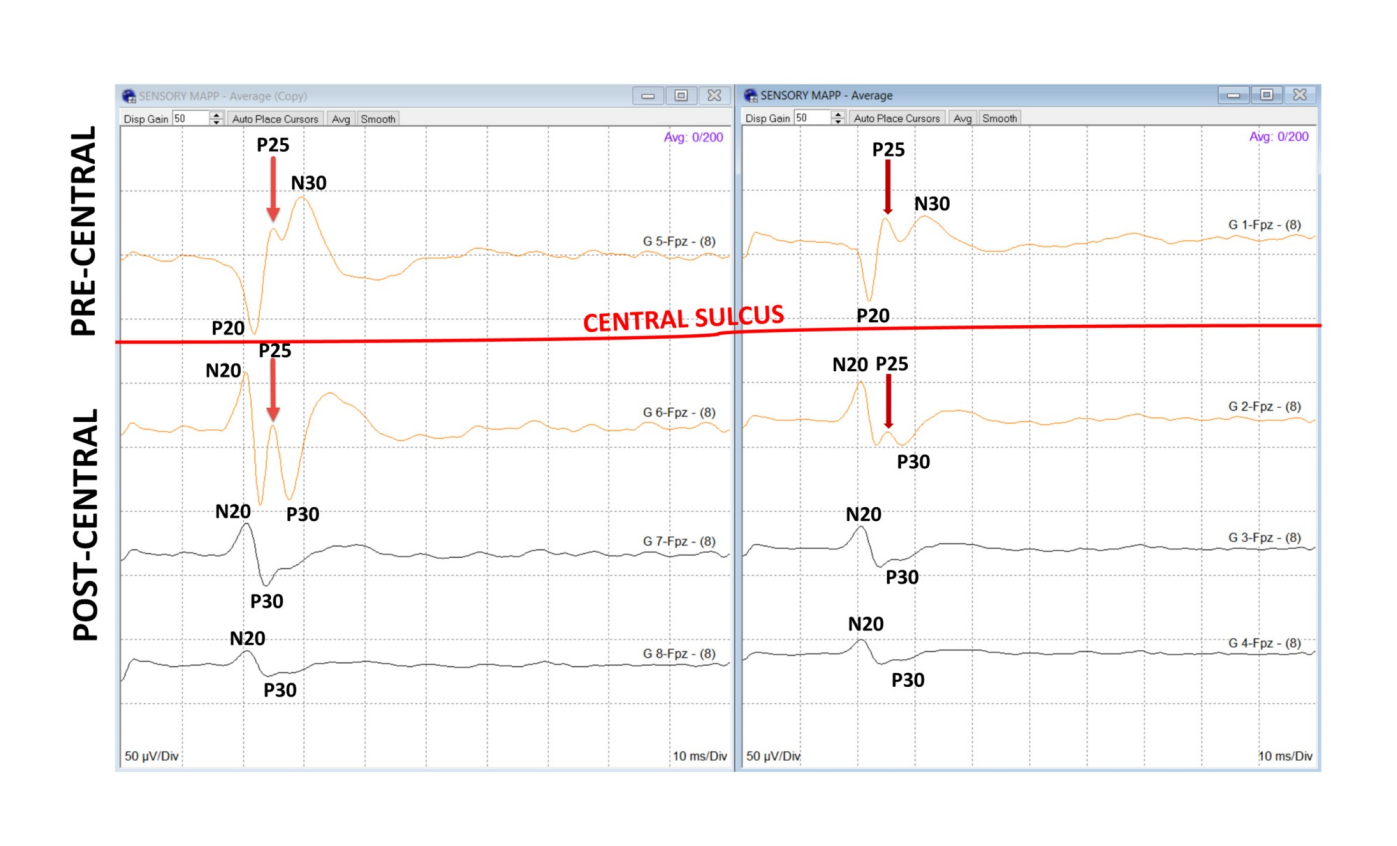
On-axis mapping. An on-axis MN sensory mapping by a 2 x 4 grid with a triphasic PR, including a P25 response. The PR is between G1/G2 and G5/G6 localizing the CS (red line). The responses from G1 and G5 are precentral, and G2, G3, G4, G7, and G8 are postcentral. MN, median nerve; PR, phase reversal; CS, central sulcus

If the grid electrode is not placed accurately over the CS, it may result in an off-axis mapping represented as the traditional dual-radial model (Figure 6) [11]. This may result in the misinterpretation of the results [1]. The on-axis mapping is performed by continuing the PR until a tangential-radial triphasic (TRT) peaks (N20/P25/P30) is recorded from the postcentral gyrus (Figure 7). The TRT cortical SSEP model generates two dipoles. The first tangential dipole generates the N20 and P20 peaks across the CS, and the second dipole generates a radially oriented P25 peak in the CS [8]. Depending on the surgery, the use of IONM with accurate CsM can offer greater accuracy of the identification of the CS and somatosensory cortex. This may decrease with varying degrees of postoperative neurological deficits. A false negative interpretation may occur if the grid contacts are above a large tumor and do not record any responses.

Other mapping techniques such as fMRI and MRI exist; however, they are not as portable as CsM. These methods also may not be as economical or practical for surgical use. Some possible pathologies that may require CsM include cerebral aneurysm, AVM, and intracranial tumors [12-13].

## Conclusions

Intraoperative neurophysiological sensory cortical mapping with the N20/P20 or N20/P25/P30 PR of the SSEPs is a safe, reliable, helpful technique to identify the CS. It provides a better understanding of the spatial location and functionality of the cortical region.

The experience of the neurophysiologist and the operating surgeon involved is a crucial factor is the successful cortical mapping. The cortical mapping helps the surgeon intraoperatively to identify the CS and differentiate the sensory and motor cortex. Multimodality cortical mapping should be performed when the motor function is at risk and may result in any postoperative neurological deficits.
